# Phase 1b study of galunisertib and ramucirumab in patients with advanced hepatocellular carcinoma

**DOI:** 10.1002/cam4.3880

**Published:** 2021-04-02

**Authors:** James J. Harding, Richard K. Do, Amin Yaqubie, Ann Cleverly, Yumin Zhao, Ivelina Gueorguieva, Michael Lahn, Karim A. Benhadji, Robin K. Kelley, Ghassan K. Abou‐Alfa

**Affiliations:** ^1^ Memorial Sloan Kettering Cancer Center New York NY USA; ^2^ Weill Cornell Medical College New York NY USA; ^3^ Elli Lilly and Company Indianapolis IN USA; ^4^ Helen Diller Cancer Center University of California San Francisco San Francisco CA USA

**Keywords:** galunisertib, HCC, hepatocellular carcinoma, Phase 1, ramucirumab, TGF‐β, VEGF

## Abstract

**Background:**

Preclinical data suggest that vascular endothelial growth factor (VEGF) and transforming growth factor (TGF)‐β signaling interact to stimulate angiogenesis and suppress antitumor immune responses. Thus, combined inhibition of both pathways may offer greater antitumor activity compared with VEGF‐targeted antiangiogenic monotherapy against hepatocellular carcinoma (HCC).

**Methods:**

This is a multicenter, open‐label, phase 1b study of galunisertib, an inhibitor of TGF‐β receptor 1, and ramucirumab, an anti‐VEGF receptor 2 antibody, in patients with advanced HCC aiming to define the maximum tolerated dose (MTD). Secondary objectives included safety, pharmacokinetics (PK), antitumor efficacy, and plasma alpha‐fetoprotein and TGF‐β kinetics. Dose escalation employed a 3 + 3 design. Patients received galunisertib at 80 mg (cohort 1) or 150 mg (cohort 2) orally twice a day on days 1–14 of a 28‐day cycle combined with ramucirumab 8 mg/kg intravenously every 2 weeks.

**Results:**

Eight patients were enrolled: three in cohort 1 and five in cohort 2 (two patients were unevaluable due to rapid disease progression and replaced). No dose‐limiting toxicities were observed. Treatment‐related adverse events (AEs) of any grade in ≥2 patients included nausea (25%) and vomiting (25%). There was one Grade 3 treatment‐related AE, a cerebrovascular accident possibly related to ramucirumab. Galunisertib exposure was dose‐proportional and not affected by ramucirumab. The RECIST version 1.1 objective response rate and disease control rate were 0% and 12.5%, respectively.

**Conclusion:**

Combination therapy was safe and tolerable and displayed favorable PK. The MTD was established at galunisertib at 150 mg orally twice a day and ramucirumab 8 mg/kg intravenously every 2 weeks. The results do not support the preclinical hypothesis that blocking TGFβ signaling enhances efficacy of VEGF‐targeted therapy; thus further clinical development was halted for the combination of galunisertib and ramucirumab.

## Graphical abstract

The combination of galunisertib (up to 150 mg twice as day) and ramucirumab 8 mg/kg intravenously every 2 weeks (standard dose) was safe and tolerable and displayed favorable pharmacokinetics in patients with advanced hepatocellular carcinoma. The results do not support the preclinical hypothesis that blocking transforming growth factor‐beta signaling enhances efficacy of VEGF‐targeted therapy; thus further clinical development was halted for the combination of galunisertib and ramucirumab.

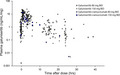

## 1 INTRODUCTION

Vascular endothelial growth factor (VEGF) is a critical driver of hepatocarcinogenesis and drugging the VEGF signaling axis represents a clear vulnerability in preclinical models of HCC.^1^ In the clinic, numerous tyrosine kinases^2^, ^3^, ^4^, ^5^ and monoclonal antibodies^6^, ^7^ that block VEGF or its receptors (VEGF‐R1‐3) have been shown to improve overall survival of patients with advanced hepatocellular carcinoma (HCC) in both the first‐ and second‐line setting. More recently, studies have indicated that antiangiogenic treatment remodels the immune microenvironment by normalizing proinflammatory cytokines, activating antigen‐presenting cells, polarizing tumor‐associated macrophages, and enhancing T cell trafficking.^8^ Such diverse effects appear to reverse immunosuppressive signals in the tumor microenvironment and may augment an anticancer immune response, particularly when paired with an immune checkpoint inhibitor.^9^ Subsequent to the study presented herein, the combination of atezolizumab, an anti‐PD‐L1 antibody, and bevacizumab, a monoclonal antibody to VEGF, were found to offer superior overall survival to sorafenib in a randomized phase 3 study in treatment‐naive advanced HCC.^9^

Ramucirumab, a fully IgG1 monoclonal antibody VEGF‐R2, has been evaluated in the second‐line setting, and did not affect survival in an unselected HCC population; improved outcomes were only evident among a poor prognosis alpha‐fetoprotein (AFP)‐high subgroup (≥400 ng/ml).^7^ In a follow‐up randomized, placebo controlled, phase 3 study (REACH‐2), restricted to AFP‐high (≥400 ng/ml) HCC patients, single agent ramucirumab led to a RECIST v1.1 objective response rate (ORR) of 5%, disease control rate of 59.9%, progression‐free survival of 2.8 months and overall survival of 8.5 months.^6^ In contrast, patients on best supportive care had an ORR of 1%, disease control rate of 38.9%, progression‐free survival of 1.6 months and overall survival of 7.3 months. Given the statistically significant improvement in disease control, progression‐free and overall survival over placebo, ramucirumab was approved after sorafenib failure or intolerance in AFP high HCC patients. Ramucirumab represents a potential backbone of antiangiogenic therapy to which other novel agents might be rationally added to improve outcomes for HCC patients.

A complementary signaling molecule to VEGF that contributes to progression of some HCCs is transforming growth factor‐beta (TGF‐β), a proinflammatory/profibrotic cytokine. TGF‐β is elevated in a subset of HCCs, serves to increase neovascularization, promote immunosuppression and immune escape, and increase migration and invasion.^10^ Indeed, HCCs with evidence of TGF‐β activation also behave more aggressively and portend a worse prognosis than those not mediated by TGF‐β.^11^, ^12^

Galunisertib, an oral, small molecule inhibitor of TGF‐β‐receptor I (ALK5), interferes with TGF‐β signaling and is deleterious to HCC in vitro and in vivo.^13^ The agent inhibits HCC growth, reduces tumor vascularity, impairs HCC motility and invasiveness, repairs fibrosis, and enhances the local immune response.^14^, ^15^, ^16^ Phase 1 and 2 studies of galunisertib monotherapy in patients with advanced HCC confirmed a favorable safety profile, which is non‐overlapping with antiangiogenic agents, and a modest degree of clinical benefit via cytostatic disease control.^17^ Preclinical data support the hypothesis that targeting both VEGF‐R2 and TGF‐β may offer greater antitumor activity by synergistically reducing angiogenesis and, at the same time, further enhancing the immune response against malignancy.^18^, ^19^ Thus, a combinatorial approach is worthy of investigation and pairing ramucirumab with galunisertib is reasonable in the context of a phase 1b study.

## 2 METHODS

### 2.1 Study design

This was a multicenter, open‐label, prospective, phase 1b study of galunisertib and ramucirumab. The primary objective was to define the maximum tolerated dose (MTD) of the combination in advanced HCC patients. Secondary objectives included safety, tolerability, pharmacokinetics (PK), estimation of efficacy, and plasma AFP and TGF‐β kinetics. The phase 1b (Part D) study reported herein was conducted under an umbrella protocol (clinicaltrials.gov, NCT01246986) that also evaluated galunisertib as a single agent (Part A and B) and in combination with sorafenib (Part C); the results for those arms have been reported previously.^17^, ^20^

Eligible patients received galunisertib at 80 mg (Cohort 1) or 150 mg (Cohort 2) orally twice a day (BID) from day 1 to 14 of a 28‐day cycle and ramucirumab at 8 mg/kg as an intravenous (IV) infusion on days 1 and 15 of every cycle. Patients continued treatment until they developed intolerable toxicity or progression of disease or withdrew from the study.

All patients provided written informed consent prior to study enrollment and the clinical trial was reviewed, approved, and monitored by the local institutional review and privacy boards. The study was conducted in accordance with the International Conference on Harmonization E6 Guidelines for Good Clinical Practice.

### 2.2 Patient population

Eligible patients were ≥18 years of age with a histologic diagnosis of HCC, were not amenable to curative surgical resection, and had adequate organ function as indicated by Child‐Pugh A (CP‐A) score. All patients had measurable disease per Response Evaluation Criteria In Solid Tumor Version 1.1 (RECIST v 1.1).^21^ Patients were required to have had progression of disease on, intolerance to, or declined prior sorafenib, the only front‐line standard of care at the time of study conduct. Any number of prior systemic therapies were allowed. Key exclusion criteria included: other histologies including mixed HCC‐cholangiocarcinoma and fibrolamellar carcinoma, clinically relevant ascites or encephalopathy, prior liver transplant, uncontrolled hypertension (≥150/90 mmHg despite standard medical management), moderate or severe cardiac disease; severe proteinuria (≥1000 mg in 24‐h period), major surgery within 28 days, gastrointestinal hemorrhage within 3 months, thrombotic event within 6 months of enrollment, and therapeutic anticoagulation.

### 2.3 Study procedures and clinical assessment

Patients were evaluated on day 1 and day 15 of each 28‐day cycle by office visit, laboratories, and physical examination. All adverse events (AEs) were recorded per the NCI CTCAE version 4.0.^22^ Treatment response was assessed using liver three‐phase computed tomography (CT) with chest and pelvis, or a chest CT and magnetic resonance imaging of the abdomen and pelvis, at baseline and then every 6 weeks for the duration of study using RECIST 1.1 criteria.^21^

### 2.4 Pharmacokinetics

Blood samples for measuring galunisertib concentrations were collected pre‐dose and 1.5–3 h post‐dose on day 1 of cycle 1; pre‐dose and 0.5, 2, 3, and 6 h post‐dose on day 14 of cycle 1; pre‐dose and 1.5–3 h post‐dose on day 1 of cycle 2; pre‐dose and 0.5–2 and 3–5 h post‐dose on day 14 of cycle 2, and pre‐dose on day 1 of cycles 3 and 4. Galunisertib concentrations were determined by an established and validated chromatography‐mass spectrometry method (Elli Lilly).^23^, ^24^ AFP and TGF‐β1 levels in plasma were measured every 2 weeks and analyzed by enzyme‐linked immunosorbent assay (DB100B; R&D Systems).

### 2.5 Dose escalation criteria and definition

Dose escalation was driven by safety using the standard 3 + 3 design. Both cohorts planned to enroll a minimum of three patients. If one patient in either dose level experienced a dose‐limiting toxicity (DLT) within the first 28‐day cycle of galunisertib and ramucirumab, then up to three additional patients were enrolled at that dose level. If zero of three or one of six patients experienced a DLT, cohort escalation would continue until the last predefined cohort (i.e. MTD). If a DLT was observed in two or more patients at either dose level in the first cycle, dose escalation would cease, and either the previous dose level would be declared the MTD or the combination would be deemed intolerable. Intra‐patient dose escalation was not allowed. Additional enrollment of up to 15 patients at the MTD was permitted to further explore safety.

A DLT was defined as combination treatment‐related AE during cycle 1 including CTCAE Grade ≥ 3 nonhematological toxicity, Grade ≥ 4 hematological toxicity of >5 days duration, febrile neutropenia, or Grade 3 or 4 thrombocytopenia with bleeding. Notable DLT exceptions were for nausea, vomiting, constipation, diarrhea, or electrolyte disturbances that could be controlled within 72 h by supportive measures, transient (<7 days) Grade 3 AST and ALT abnormalities without evidence of hepatic injury, and controllable hypertension.

### 2.6 Biostatistics

All data were tabulated and reported using descriptive statistics. The primary objective of the study was determination of the MTD, which was defined as the highest tested dose that had <33% probability of causing a DLT during cycle 1. Nonlinear mixed‐effect modeling was used to estimate the population pharmacokinetic parameters of galunisertib. Antitumor efficacy was determined by RECIST v1.1 and modified RECIST and confirmed by central independent review. The ORR was defined as the proportion of all patients that attained a confirmed complete response (CR) or partial response (PR). The disease control rate was defined as the proportion of all patients that attained a CR, PR, or stable disease (SD). All patients who received at least one dose of the study drug(s) were included in the analysis for safety and efficacy.

## 3 RESULTS

### 3.1 Patient disposition

The study enrolled a total of eight patients from August 2015 to July 2016: three in cohort 1 and five patients in cohort 2. All patients received at least one dose of study drug and thus all were included in the analysis of both safety and efficacy. At data cutoff in June 2019, the primary reason for study discontinuation was death related to disease progression (six), hepatic failure in the follow‐up period unrelated to study drug (one), and loss to follow‐up (one).

### 3.2 Patient demographics

The characteristics of the eight patients enrolled on the study are summarized in Table 1. Patients were mostly Asian (6, 75%) and all were male (8, 100%); median age was 59 years (range 47–83) and excellent performance status (7 [87.5%] had ECOG PS 0). Seven of eight patients had virally mediated HCC (five HBV (Hepatitis B virus), two HCV (Hepatitis C virus)) and all patients had intact hepatic function according to CP‐A score. Patients had advanced disease of mostly Barcelona Clinic Liver Cancer Stage C (7, 87.5%), and a significant proportion had portal vein involvement (3, 37.5%) and AFP ≥400 ng/ml (6, 75%), and had received prior sorafenib treatment (5, 62.5%). Mean baseline serum TGF‐β concentration was 3008 ± 2069 (standard deviation).

**TABLE 1:** Patient demographics (n = 8). Categorical data are presented as n (%) and continuous as mean (SD) unless otherwise indicated

	Cohort 1 Galunisertib 160 mg BID n = 3	Cohort 2 Galunisertib 300 mg BID n = 5	Total n = 8
Sex, male	3 (100%)	5 (100%)	8 (100%)
Age, median (range)	48 (47–67)	59 (56–83)	58.5 (47–83)
Race			
Asian	2 (66.7%)	4 (80%)	6 (75%)
Black	0	1 (20%)	1 (12.5%)
Unknown	1 (33.3%)	0	1 (12.5%)
ECOG performance status			
0	3 (100%)	4 (80%)	7 (87.5%)
1	0	1 (20%)	1 (12.5%)
Etiologic factors			
HBV	1 (33.3%)	4 (80%)	5 (62.5%)
HCV	2 (66.7%)	0	2 (25.0%)
Non‐viral	0	1 (20%)	1 (12.5%)
Child‐Pugh score			
A5	1 (33.3%)	3 (60.0%)	4 (50.0%)
A6	2 (66.7%)	2 (40.0%)	4 (50.0%)
BCLC stage			
A	0	1 (20%)	1 (12.5%)
B	0	0	0
C	3 (100%)	4 (80%)	7 (87.5%)
Portal vein involvement	0	3 (60%)	3 (37.5%)
Prior sorafenib	3 (100%)	2 (40%)	5 (62.5%)
AFP, ng/ml			
<200	0(0%)	2 (40%)	2 (25%)
>400	3 (100%)	3 (60%)	5 (75.0%)
TGFβ, pg/ml	2662.7 (1433.14)	3214.9 (2514.77)	3007.9 (2069.36)

Abbreviations: AFP, alpha‐fetoprotein; BCLC, Barcelona Clinic Liver Cancer; TGFβ, transforming growth factor‐beta.

### 3.3 Safety

No DLTs were observed in cohort 1 or 2. In cohort 2, one patient developed Grade 3 hepatic encephalopathy at day 10 of cycle 1, attributed to rapidly progressive HCC and unrelated to study treatment, and a second patient had clinical disease progression. An additional two patients were recruited to cohort 2 to enable collection of data over the entire DLT period.

Treatment‐emergent AEs of any grade occurring in ≥2 patients included epistaxis (4, 50%), abdominal pain (3, 37.5%), abdominal distention (3, 37.5%), nausea (2, 25%), diarrhea (2, 25%), musculoskeletal chest pain (2, 25%), peripheral edema (2, 25%), dyspnea (2, 25%), fatigue (2, 25%), and hepatic encephalopathy (2, 25%) (Table 2). Treatment‐related AEs of reported in ≥2 patients were nausea and diarrhea (two patients, 25% each).

**TABLE 2:** Any grade treatment‐emergent adverse events in HCC patients treated with galunisertib and ramucirumab

CTCAE term	Cohort 1	Cohort 2	Total
	n = 3	n = 5	n = 8 (%)
Epistaxis	3	1	4 (50)
Diarrhea	1	1	2 (25)
Chest pain	2	1	3 (37.5)
Abdominal distention	1	2	3 (37.5)
Dyspnea	1	2	3 (37.5)
Abdominal pain	0	3	3 (37.5)
Fatigue	0	3	3 (37.5)
Nausea	1	1	2 (25)
Peripheral edema	1	1	2 (25)
Hepatic encephalopathy	0	2	2 (25)
Dry mouth	1	0	1 (12.5)
Muscle weakness	1	0	1 (12.5)
Viral syndrome	1	0	1 (12.5)
Upper Respiratory Infection	1	0	1 (12.5)
Cerebrovascular accident	2	0	2 (25)
Secondary malignancy (PNET)	0	1	1 (12.5)
Blood bilirubin increase	0	1	1 (12.5)
Constipation	0	1	1 (12.5)
Dysgeusia	0	1	1 (12.5)
Cough	0	1	1 (12.5)
Nocturia	0	1	1 (12.5)
Gastrointestinal hemorrhage	0	1	1 (12.5)
Chills	0	1	1 (12.5)
Fever	0	1	1 (12.5)
Headache	0	1	1 (12.5)
Pain	0	1	1 (12.5)
Confusional state	0	1	1 (12.5)
Portal vein thrombus	0	1	1 (12.5)
Sore throat	0	1	1 (12.5)

Abbreviation: PNET, pancreatic neuroectodermal tumor.

Serious AEs of any attribution included: Grade 3 cerebrovascular accident, Grade 3 hepatic encephalopathy, Grade 5 hepatic encephalopathy, Grade 4 gastrointestinal hemorrhage, and Grade 3 pancreatic neuroendocrine tumor. The Grade 3 cerebrovascular accident occurred after the DLT period at day 4 of cycle 2 in cohort 1, was possibly related to ramucirumab, unlikely related to galunisertib, and led to permanent discontinuation of treatment. As mentioned above, the Grade 3 hepatic encephalopathy during study treatment in cohort 2 was deemed unrelated to study drug. The Grade 5 hepatic encephalopathy occurred simultaneously with a gastrointestinal hemorrhage within 33 days of cession of study treatment, and was unrelated to study drugs. In one patient, a pancreatic mass evolved over study treatment and was found by biopsy to be a low‐grade neuroendocrine tumor that was deemed unrelated to study treatment and monitored by expectant observation.

### 3.4 Pharmacokinetics

**FIGURE 1:**
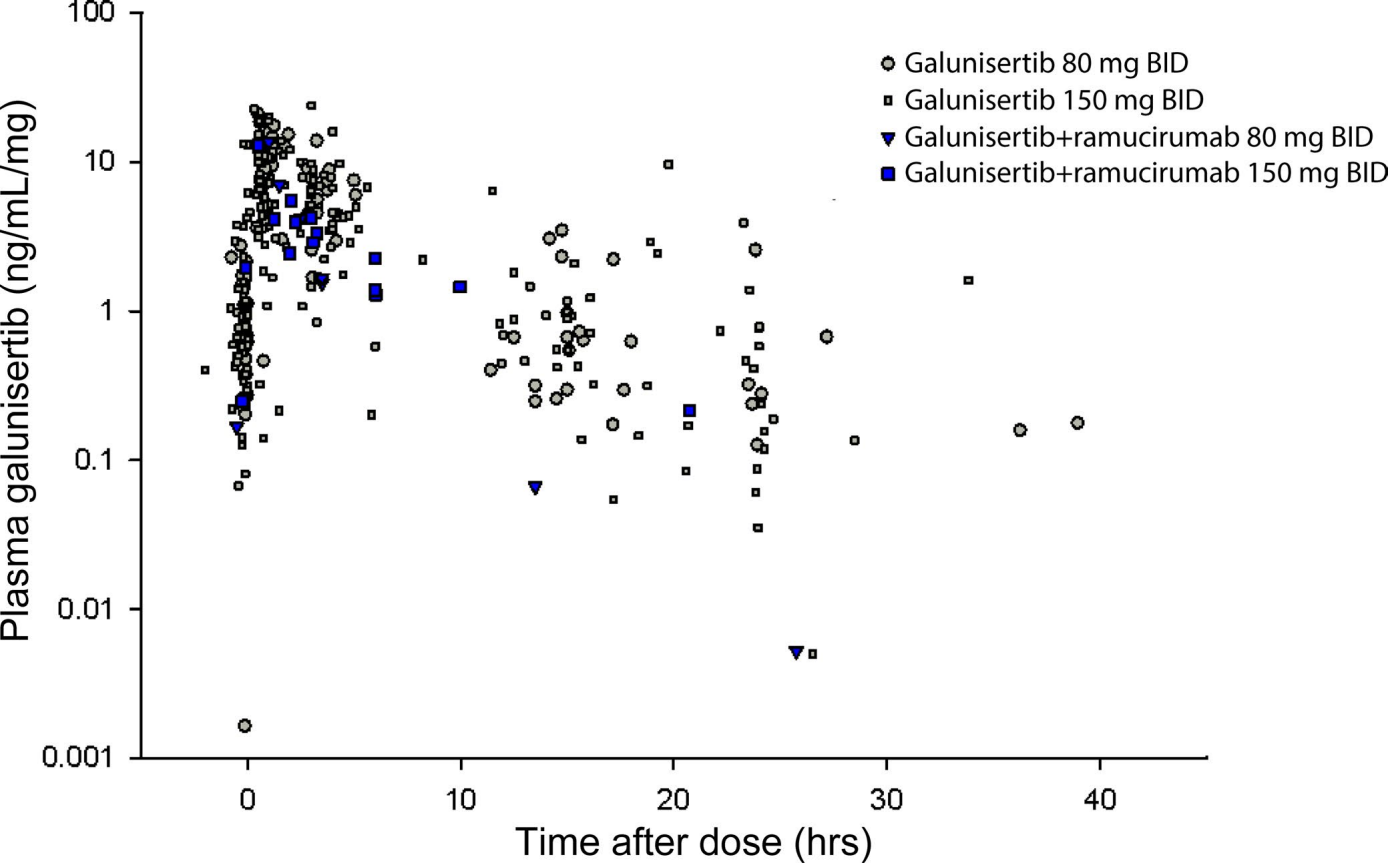
Dose‐normalized plasma galunisertib concentrations at steady state (following treatment on day 14) with monotherapy (Part A) and combination with ramucirumab (Part D)

To assess the potential impact of ramucirumab on plasma galunisertib exposures, normalized steady‐state galunisertib concentrations (area under the curve from 0–8 h on day 14 of cycles 1 and 3) from patients treated with the combination (three and four patients receiving 80 and 150 mg, respectively) were compared to those in patients treated with galunisertib monotherapy (Figure 1). Individual patient pharmacokinetic parameters could not be estimated due to incomplete time and/or dosing information. A total of 23 steady‐state plasma concentrations are presented for combination treatment. No qualitative differences were observed; galunisertib was rapidly absorbed and eliminated within 36 h.

### 3.5 Antitumor activity and biomarkers

Of eight patients enrolled, no patient attained a RECIST v1.1 complete or PR, one patient had SD, three patients had progressive disease, and four patients were not evaluable. When mRECIST was employed in preplanned protocol analysis, one patient achieved a confirmed PR (cohort 1), three had progressive disease, and four were not evaluable. The disease control rate was 12.5% by RECIST v1.1 and mRECIST.

**FIGURE 2:**
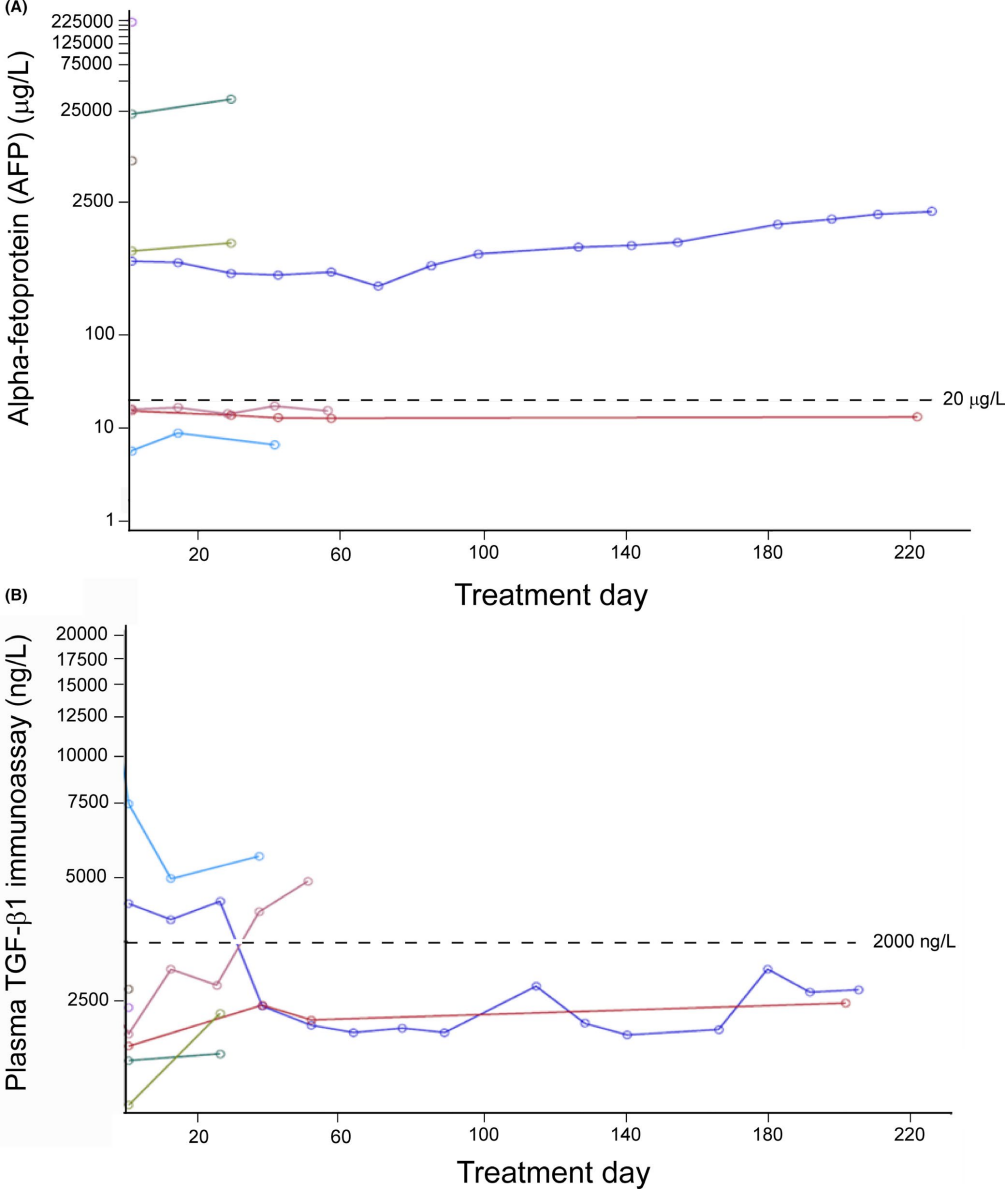
Plasma concentration of AFP (A) and TGF‐β1 (B) in patients treated with ramucirumab and galunisertib over time. Each line represents an individual patient. AFP, alpha‐fetoprotein; TGF‐β, transforming growth factor‐beta

Plasma AFP and TGF‐β were determined at baseline and over the course of treatment for all patients on study (Figure 2). No change in AFP concentrations was apparent over the period of treatment. In the one mRECIST responder that exceeded 6 months, TGF‐β concentrations decreased by nearly 50% over the course of cycle 1.

## 4 DISCUSSION

Galunisertib combined with ramucirumab was safe and tolerable in patients with advanced HCC who had intact liver function in this phase 1b study. No new safety signals were observed following combination therapy when compared with galunisertib or ramucirumab alone. Pharmacokinetic assessment suggested that ramucirumab has no impact on steady state concentrations of galunisertib, though a notable limitation was the limited sample size and incomplete patient level PK data. The MTD was established at 150 mg orally BID from day 1 to 14 with ramucirumab at 8 mg/kg administered IV on days 1 and 15 of a 28‐day cycle.

The scientific basis underling this clinical study were the preclinical observations that galunisertib might augment the antitumor activity of ramucirumab. The ORR by RECIST v1.1 was 0% and the disease control rate was 12.5% for the combination, indicating limited antitumor activity in comparison to historic controls of ramucirumab alone.^6^ These results do not support the hypothesis that blocking TGFβ signaling using galunisertib enhances the clinical efficacy of VEGF‐targeted therapy using ramucirumab in advanced HCC.

Aspects related to the trial design, such as the small sample size, dose escalation, and lack of AFP cutoff for trial entry, may of course have confounded an apparent efficacy signal. Regarding the latter, lack of AFP cutoff must be considered as one potential limitation, despite the observation that only two of eight study participants (25%) had a baseline AFP <400 ng/ml. After the completion of the study presented herein, it became clear that pre‐treatment AFP levels ≥400 ng/ml were requisite for a survival advantage to ramucirumab monotherapy in the second‐line. Differential outcomes based on pretreatment AFP suggest dependence on VEGF pathway signaling in tumors with high AFP expression. However, AFP also increases with disease stage and other aggressive clinicopathologic features, and it is unclear how this relates to any potential underlying biology and ramucirumab mechanism of action.^25^

Biologic factors may have also contributed to a lack of efficacy.^10^ For example, galunisertib interferes with ligand‐mediated signaling through TGFβ‐RI kinase activity; however, parallel signaling pathways have been shown to activate TGFβ1 gene transcripts in a kinase‐independent fashion. Subsets of gastrointestinal cancers, including HCC, are also known to inactivate TGFβ‐RI and downstream signaling components, such as SMAD2 and SMAD4, through genomic, epigenomic, and transcriptomic alterations.^26^ These observations predict that some HCCs are innately insensitive to co‐targeting, and indeed, preclinical models reported after the completion of this study using combined VEGF and TGFβ inhibition have shown variable efficacy due to such biologic heterogeneity.^27^ Alternatively, it is established that TGF‐β exhibits context dependent and differential action on tumor growth and survival—in select instances TGF‐β signaling is required for clearance of neoplasia.^10^ Although speculative, it is possible that TGF‐β inhibition might paradoxically drive tumor growth leading to worse clinical outcomes. Preclinical models and correlative analyses from clinical studies of galunisertib do not support such a notion.^28^, ^29^ Nevertheless, our data imply that pairing antiangiogenics with agents blocking TGF‐β is not a robust strategy clinically.

A critical question that remains is whether galunisertib, or agents with similar mechanisms of TGF‐βI receptor inhibition, are effective in a proportion of HCCs and whether this population can be selected based on one or more biomarkers. Prior data suggest that baseline circulating plasma AFP and TGF‐β1, AFP and TGF‐β1 response kinetics, and tumor SMAD complex activity might discriminate a sensitive HCC subset.^30^ Although AFP and TGF‐β1 were sampled over treatment on our study, the small sample size limits any definitive conclusion regarding the prognostic value of baseline plasma levels, or the predictive value of AFP or TGF‐β1 response. Furthermore, as pretreatment biopsy was not mandated due to safety considerations related to using an antiangiogenic, activity of the TGFβ axis at the tumor level could not be assessed.

Given the advent of immune checkpoint inhibitors serving as the backbone for therapy in advanced HCC patients, the favorable safety profile and TGF‐β receptor inhibition of galunisertib supports evaluating it in combination with immunotherapy. Gene expression profiling indicate an HCC subset exhibits a TGF‐β signature and this associates with a T‐cell exhausted phenotype and a high proportion of immunosuppressive T‐regulatory cells.^31^ Preclinical data indicate synergistic activity of blocking TGF‐β and checkpoint inhibitors in multiple solid tumors.^32^, ^33^, ^34^ Emerging clinical data also suggest value in co‐targeting TGF‐β and the PD‐1/PD‐L1 axis. For example, bintrafusp alfa/M7824 is an anti‐PD‐L1 antibody fused to the extracellular domain of TGF‐β receptor 2 which function as a TGF‐β “trap.” The agent was found to be safe and tolerable with anecdotes of durable responses in treatment‐refractory hepato‐pancreaticobiliary cancers on a recent phase 1 study.^34^ Several phase 2 and phase 3 studies are currently ongoing with this agent in histologic‐specific indications as well as on basket studies. Importantly, a study of galunisertib in combination with nivolumab (NCT02423343) in advanced HCC patients is currently ongoing and will further clarify the role of combined TGF‐β and PD‐1 blockade in HCC. A critical consideration, and necessary readout for such data will be tissue correlates to ultimately define a unique TGF‐β driven HCC subset that is most sensitive to such combinatorial strategies.

In summary, combination treatment was tolerable and the MTD of galunisertib was established. Due to a shifting landscape of HCC treatment and the combination's apparent lack of antitumor activity, the study was terminated and further clarification the antitumor activity of the combination was not pursued.

## CONFLICT OF INTEREST

JJH has received research support from Bristol Myers Squibb, and has received consulting fees from Eli Lilly, Exelexis, Eisai, Bristol Myers Squibb, CytomX, Imvax, and Merck. RKD and AY report no relevant relationships with commercial entities. AC, YZ, IG, and ML are employees of and own stock in Elli Lilly. KAB is an employee of and owns stock in Taiho Oncology. RKK has received personal fees from Genentech/Roche and Gilead, and research funding from Adaptimmune, Agios, AstraZeneca, Bayer, Bristol Myers Squibb, EliLilly, Exelixis, EMD Serono, Merck, Novartis, QED, Taiho, Partner Therapeutics, and Ipsen. GKA has received research support from ActaBiologica, Agios, Astra Zeneca, Bayer, Beigene, Berry Genomics, BMS, Casi, Celgene, Exelixis, Genentech/Roche, Halozyme, Incyte, Mabvax, Puma, QED, Sillajen, and Yiviva; and consulting fees from Agios, Astra Zeneca, Autem, Bayer, Beigene, Berry Genomics, Celgene, CytomX, Debio, Eisai, Eli Lilly, Exelixis, Flatiron, Genentech/Roche, Gilead, Helio, Incyte, Ipsen, Loxo, Merck, MINA, Polaris, QED, Redhill, Silenseed, Sillajen, Sobi, Therabionics, Twoxar, Vector, and Yiviva.

## AUTHOR CONTRIBUTIONS

James J. Harding contributed to study conceptualization, protocol writing, data acquisition, data analysis, and study supervision, and wrote the original draft of the manuscript. Richard K. Do contributed to data acquisition and data analysis. Amin Yaqubie contributed to data acquisition and data analysis. Ann Cleverly, Yumin Zhao, Ivelina Gueorguieva, Michael Lahn, Karim A. Benhadji, Robin K. Kelley, and Ghassan K. Abou‐Alfa contributed to study conceptualization, protocol writing, data acquisition, data analysis, and study supervision. All authors contributed to manuscript development and reviewed and approved the final version.

## ETHICAL APPROVAL

All patients provided written informed consent prior to study enrollment and the clinical trial was reviewed, approved, and monitored by the local institutional review and privacy boards. The study was conducted in accordance with the International Conference on Harmonization E6 Guidelines for Good Clinical Practice.

## Data Availability

Raw data are available upon request.
